# Fidelity of Automatic Speech Processing for Adult and Child Talker Classifications

**DOI:** 10.1371/journal.pone.0160588

**Published:** 2016-08-16

**Authors:** Mark VanDam, Noah H. Silbert

**Affiliations:** 1 Department of Speech & Hearing Sciences, Elson S. Floyd College of Medicine, Washington State University, Spokane, Washington, United States of America; 2 Spokane Hearing Oral Program of Excellence (HOPE), Spokane, Washington, United States of America; 3 Department of Communication Sciences & Disorders, University of Cincinnati, Cincinnati, Ohio, United States of America; University of Kent, UNITED KINGDOM

## Abstract

Automatic speech processing (ASP) has recently been applied to very large datasets of naturalistically collected, daylong recordings of child speech via an audio recorder worn by young children. The system developed by the LENA Research Foundation analyzes children's speech for research and clinical purposes, with special focus on of identifying and tagging family speech dynamics and the at-home acoustic environment from the auditory perspective of the child. A primary issue for researchers, clinicians, and families using the Language ENvironment Analysis (LENA) system is to what degree the segment labels are valid. This classification study evaluates the performance of the computer ASP output against 23 trained human judges who made about 53,000 judgements of classification of segments tagged by the LENA ASP. Results indicate performance consistent with modern ASP such as those using HMM methods, with acoustic characteristics of fundamental frequency and segment duration most important for both human and machine classifications. Results are likely to be important for interpreting and improving ASP output.

## Introduction

Automatic speech processing (ASP) technology has been used increasingly in a wide variety of scientific and practical applications. Preliminary work in speech recognition began in the 1960s, with talker-independent automatic speech recognition and ASP work gaining a foothold by in the 1980s [1] and the first attempt at child speech not coming until the mid-1990s [2,3]. The majority of the literature considers ASP as it is applied to healthy adult speech, although there has been some attention to the application of ASP with child speech [4,5,6,7] and disordered populations [8,9]. One ASP system used with children and disordered children is the Language ENvironment Analysis (LENA; LENA Research Foundation, Boulder, CO).

The LENA system is the first and, to date, only of its kind to allow for massive-scale naturalistic speech data to be collected and analyzed with ASP methods.

Researchers utilize LENA to analyze patterns of typical development [10,11,12,13], autism spectrum disorders [14,15,16,17], childhood hearing loss [18,19,20], Downs syndrome [21], the consequences of premature birth [22,23], the impact of television viewing [12,24,25,26], and classroom communication [27].

### Description of the device

The LENA system consists of a body-worn audio recorder designed to be worn unobtrusively on the body of a child and proprietary ASP processing software [13]. The system hardware is designed to collect unprocessed whole-day audio recordings (up to 16 h) [28]. After the audio is uploaded to a computer, LENA software processes the audio off-line. The result of processing is a time-aligned record of the segmentation at centisecond resolution and assignment of one of about 60 apriori labels to each segment, providing details of the auditory environment of the child wearing the recorder. The labels are broadly divided into environmental and live human-vocal categories. The environmental labels include tags such as *noise*, *electronic media*, *silence*, and *uncertain/fuzzy*. The live human-vocal category includes labels such as *key-child vegetative*, *key-child speech-like*, *adult male*, *adult female*, and *other child*.

### Description of the technology

The ASP processing used by the LENA is a set of algorithms designed to assess child and family speech [29]. The system relies on standard, modern ASP methods, using Gaussian mixture and hidden Markov models to segment and assign labels [13,28,30]. It uses an optimized, dynamically programmed searching algorithm to compare acoustic templates with diphones in the observed segment to achieve a maximum likelihood match, ultimately assigning one label to that segment [31,32,33].

### Reliability of the technology

Empirical studies on the reliability of the LENA labels and adult word counts compared to human coders has been reported for typically-developing (TD) children learning English [14,15,24,28,34], Spanish [35], and French [36]. In these reports, 82% of segments coded by humans as *adult* were similarly coded by machine; 73–76% of segments coded by humans as *child* were similarly coded by machine. For those segments the ASP labeled as *adult* and *child*, humans similarly labeled segments 68% and 64–70%, respectively.

One recent, detailed study looked at the validation of the LENA machine labels compared with human transcriptions in a dataset representing 94 family recordings of children 2–48 months of age [33]. They found greater than 72% machine-human agreement in segments identified as "clear key child." This finding was shown to be similar to the 76% agreement the same research group found in a subset sample of 70 family recordings with children 2–36 months of age [30]. Xu and colleagues [33] further analyzed a subset of about 2374 child utterances for phonetic content validity to assess the robustness of the machine classifier. Phonetic units in the child utterances were analyzed by open source Sphinx speech recognition software based on well-pronounced adult speech models. The machine recognizer segmented and assigned one of 46 labels in categories of consonant, vowel, nonspeech, and silence. Human transcribers coded the same child utterances, and the machine and human transcriptions were then compared. The correlations between machine and human coders' average count of consonants and vowels per utterance were high, 0.85 and 0.82, respectively. The machine recognition algorithms significantly underestimated the total number of vowels and consonants, but overall the results gave robust reliability estimates of the ASP software in this domain.

### Research questions

A persistent issue of ASP, especially as it is applied to natural speech, child speech, and disordered speech, is the reliability of its labeling. The goal of this report is to provide an empirical examination of LENA ASP output using a large sample of machine-labeled segments of interest to speech and language work evaluated by human judges comprising a generalizable gold standard with which to compare machine labels. The specific research questions are as follows:

1. How does LENA machine ASP speaker classification performance compare with human coders? In particular, how do the machine labels for *target child*, *adult female*, and *adult male* compare with human judge assessments?
2. How are LENA machine classification errors organized? That is, are certain types of errors or confusions more common than others?
3. How are acoustic signal characteristics known to be important for speech (duration, fundamental frequency, and amplitude) associated with human and LENA machine classification performance?

## Materials and Method

### Participants

Twenty-three judges evaluated the same stimuli to assess inter-judge reliability. All judges were formally trained in speech and hearing sciences and had familiarity with judgement testing procedures. All but two judges were female.

### Materials

Twenty-six recordings, one recording from each of 26 families, were used for this study. All families lived together full-time, and all children were typically-developing. Demographic factors of the families such as socio-economic status, ethnicity, or race were not examined in the present work. The recordings from each family were collected and organized based on the age of the child wearing the recording device, averaging 29.1 months (*SD* = 2.7 months). Twenty-three independent judges evaluated the recordings.

Each daylong acoustic recording was analyzed with the LENA system. To obtain the daylong audio recordings, families were given a shirt with a custom chest pocket designed to hold the small, self-contained audio recorder unobtrusively on the child's chest. The family was instructed to turn the recorder on in the morning, place it into the chest pocket, and turn it off in the evening. Families recorded on days that were typical (not the child's birthday, for example) and on a day when family members including the father or an adult male were present. Raw recordings were uploaded to a computer and processed offline using LENA software. Processed recordings generated a daylong audio file (16-bit, 16 kHz, lossless PCM, WAV format) and an XML-coded record of the segmentation onset/offset points with the segment label for every segment. The present work does not consider the segmentation accuracy of the machine algorithms. It assumed that the result of the segmentation procedures are sufficient to evaluate the labeling procedures described here.

The LENA software identifies the onset and offset times of segments determined probabilistically of being live vocal segments belonging to an adult female (*FAN* segments), an adult male (*MAN* segments), or the child wearing the recording device (*CHN* segments). For each of the 78 recordings selected, three recorded talkers in each of 26 families, 30 segments labeled as *FAN*, *MAN*, and *CHN* were excised from the daylong recording in the following manner. For each of the three talker labels of interest, the total number of segments with that label for that recording were determined, then divided by 30 to yield an integer value, *n*. Using a custom matlab script, each *n*th instance of that label was then excised from the raw, daylong recording and stored as an individual computer sound file. This distribution was used here to insure a relatively even spread of talker segments throughout the daylong recording and avoiding over- or under sampling from certain environmental situations (e.g., bath or meal times), times of (vocal) fatigue such as later in the day, or contextual variability (e.g., regularities of family members, events, or conversations topics). Thus, stimuli consisted of 30 audio segments from each of three talker labels (*FAN*, *MAN*, *CHN*) collected from 26 family recordings totaling 2340 unique audio files. There were a total of 53820 stimuli presentations, 17940 each from the machine-classified categories of *adult-female*, *adult-male*, and *target-child*.

### Ethics Statement

This study was specifically approved by the Washington State University Institutional Review Board. Information about the experiment was provided and written informed consent was collected prior to participation by both the families contributing audio recordings and the judges. In terms of the minors/children in the study, written informed consent was obtained from the parents on behalf of minors/children prior to participation.

### Acoustic characteristics of the stimuli

Acoustic characteristics known to be important to speech and language include duration, *f*_0_, *f*_0_ trajectory, amplitude, and amplitude variability over time [37,38]. Acoustic characteristics of the stimuli are given in Table 1 for segment duration, *f*_0_, and RMS (relative) amplitude. Descriptive statistics for the pooled stimuli set, and for each grouping designation, FAN, MAN, and CHN segments. Following previous studies that have examined the relationship between classifications and the acoustic factors associated with those decisions [14], here we examine the relationship between ten similar apriori acoustic features and the classification decisions of the judges. For this report, the ten features, shown in Table 2 below, give a first approximation of the underlying acoustic signal information that drives classification decisions, including possible differences between human and machine processes.

**Table 1:** Descriptive statistics of acoustic features of the stimuli.

Acoustic Feature	All-pooled (n = 2340): M (SD) range	FAN (n = 780): M (SD) range	MAN (n = 780): M (SD) range	CHN (n = 780): M (SD) range
segment duration (ms)	1154 (698) 80–9220	1360 (787) 600–9220	1329 (621) 600–7820	769 (484) 81–3680
*f*_0_ (Hz)	254 (82) 80–578	253 (59) 87–543	185 (60) 80–578	374 (90) 142–764
RMS (digital full-scale = -1.0-+1.0	.081 (.063) 0-.310	.059 (.042) 0-.259	.055 (.044) 0-.240	.129 (.068) 0-.310

**Table 2:** Acoustic Features and Descriptions.

Acoustic Feature	Description
phrase duration (ms)	phrase segment duration
mean *f*_0_ (Hz)	mean *f*_0_
minimum *f*_0_ (Hz)	minimum *f*_0_
maximum *f*_0_ (Hz)	maximum *f*_0_
rising *f*_0_ (Hz)	max *f*_0_ follows min f_0_ in phrase
falling *f*_0_ (Hz)	min *f*_0_ follows max *f*_0_ in phrase
mean RMS (dB)	mean RMS amplitude
rising amplitude (dB)	max amp follows min amp in phrase
falling amplitude (dB)	min amp follows max amp in phrase
amplitude modulation (dB)	SD of amplitude in phrase

### Procedure

Written informed consent was obtained from all participants prior to data collection. The 23 judges evaluated the same 2340 stimuli. A session consisted of the judge evaluating all the recordings from one age group, totaling 2340 decisions. Prior to each session, a custom matlab script randomized the presentation order of all stimuli for that session.

Participants were instructed to listen to each stimulus and select from *1-child*, *2-mother*, *3-father*, or *4-other* by entering the number from a standard keyboard. Judges could replay the audio stimulus an unlimited number of times, and session *percent complete* was shown in real time. After several practice trials, stimuli were presented to the judges via a nearfield monitor loudspeaker (model 8030A, Genelec, Iisalmi, Finland) adjusted by the judge to a comfortable listening level in quiet, sound-treated room. There are two notable aspects of the task. First, judges were given a four-alternative forced-choice (4AFC) task but the possible response label *other* ASP was not an actual stimulus as labeled by the LENA software. That is, only three actual LENA labels were evaluated, *FAN*, *MAN* and *CHN*, but there were four alternatives possible for the human judges to label. Second, the labels used by the LENA ASP (*FAN*, *MAN*, *CHN*) are not strict analogs offered in the forced-choice task (*mother*, *father*, *child*) to the judges. That is, the tacit task of the ASP was to assign a nominal label corresponding to an adult female (i.e., *FAN*), to an adult male (i.e., *MAN*), or to the child wearing the recorder (i.e., *CHN*), while the task of the human judge demanded identification of *mother*, *father*, and *child*. There is no guarantee that an adult woman, for example, would be unambiguously also a mother. It was assumed that there is a high correspondence between any *FAN* label, for instance, and that person being the actual mother of the child wearing the recorder. In the event that this assumption is not borne out, however, it is unlikely to have a material effect on the overall goal of estimating language learning from a child’s perspective whether the adult female, for example, is in fact that child’s actual mother or another adult female within auditory range of the child. Judges were given short breaks as needed and completed the task in 90–200 minutes. No feedback was given to the judges, and data acquisition was controlled via the custom matlab script. A short debriefing followed participation.

### Data analysis and statistical approach

In order to assess the relationship between ASP and human coder judgments, Fleiss’ kappa and Cohen’s kappa reliability coefficients were calculated. Fleiss’ kappa provides an overall measure of agreement between the ASP system and all of the human coders. Cohen’s kappa provides a separate measure of agreement between the ASP system and each human coder. Because the human coders had the option to label a stimulus *other*, while all tokens were coded *father*, *mother*, or *child* by the ASP system, the *other*-coded tokens were excluded from calculation of Fleiss’ kappa and Cohen’s kappa. To mitigate the effects of any deviation between the ASP and human coders’ classifications, weighted kappa coefficients are also reported. The weighted kappa coefficients are calculated as the raw kappa coefficients multiplied by the proportion of *father*, *mother*, or *child* judgments (i.e., one minus the proportion of *other* responses).

The patterns of agreement and disagreement are analyzed informally by analysis of a matrix indicating the proportions of tokens given each combination of classification by the ASP system and the human coders.

Classification trees were used to analyze the relationship between the acoustic data and the ASP and human coder judgments, using the rpart and rpart.plot packages in R [39,40,41]. The classification tree employs an iterative procedure in which, at each stage, each acoustic variable is partitioned to find the best criterion, and the best partition for the best variable is retained, where best is defined with respect to goodness of fit between the model and the input judgments (i.e., how closely the tree’s classifications match either the ASP system’s or the human coders’ judgments). This procedure repeats until additional partitions of the acoustic variables do not provide statistically useful increases in the goodness of fit. Data are available without restriction at Harvard Dataverse (V1) via the following URL: http://dx.doi.org/10.7910/DVN/7CW9KO.

## Results

Descriptive aggregate values of responses are given for all categories in Table 3. Valid responses were collected for 99.91% (53772 of 53820) of stimuli presented to judges, likely due to response entry errors during testing.

**Table 3:** Cross-classification Totals.

	*child*	*mother*	*father*	*other*
**ASP *child***	15338	3907	571	1630
**ASP *mother***	714	10714	3082	1911
**ASP *father***	247	1399	10760	3499

**Fig 1:**
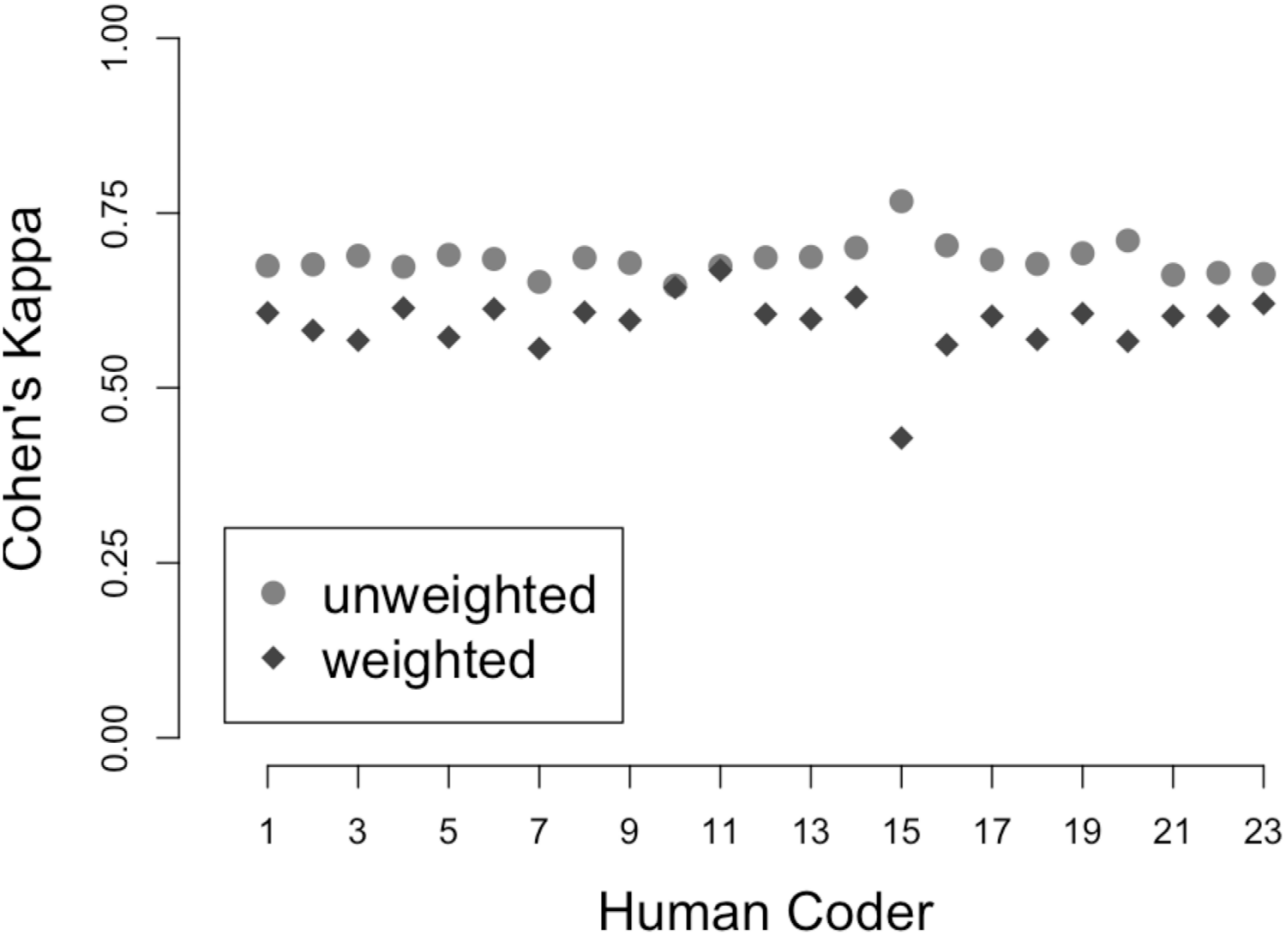
Weighted and unweighted Cohen’s kappa for each human coder. The abscissa indicates human coder index. The ordinate indicates Cohen’s kappa values. Circles indicate unweighted Cohen’s kappa coefficients. Diamonds indicate coefficients weighted by the proportion of *father*, *mother*, or *child* judgments for each coder.

Fleiss’ kappa for the full data set of human coders was 0.79. Only 13% of human coder judgments were *other*, so the weighted Fleiss’ kappa was 0.79 × 0.87 = 0.68. Both values indicate a high degree of agreement among the human coders. Fig 1 shows the unweighted (circles) and weighted (diamonds) Cohen’s kappa coefficients for each human coder. The mean unweighted Cohen’s kappa was 0.68 (min: 0.65, max: 0.77), while the mean weighted coefficient was 0.59 (min: 0.43, max: 0.67). Excepting coder 15, the level of agreement between the ASP system and the human coders was consistent both between the machine and any individual human judge and between all individual human judges, with a significant correlation between the weighted and unweighted decisions (*r* = .36, *p* < .05).

Table 4 provides the proportions of each combination of ASP and human coder classifications across for the full data set, with ASP classifications given in the rows and human coder classifications in the columns. As suggested by the high kappa coefficients presented above, most tokens were classified the same by the ASP system and the human coders. It is also clear that disagreements between the ASP system and human coders was not random. Most disagreements occurred between either *child* and *mother* judgments or between *mother* and *father* judgments, with a pronounced asymmetry between these types of disagreements.

**Table 4:** Cross-classification Proportions.

	*child*	*mother*	*father*	*other*
**ASP *child***	0.285	0.072	0.010	0.030
**ASP *mother***	0.013	0.199	0.057	0.036
**ASP *father***	0.004	0.026	0.200	0.065

With tokens classified by human coders classified as *child*, when the ASP system disagreed, it was far more likely to classify a token as *mother* than *father* (leftmost column). However, with tokens classified by human coders as *mother*, when the ASP system disagreed, it was far more likely to classify a token as *father* than *child* (second column from left). With tokens classified by humans as *father*, the ASP system was also more likely to disagree by classifying a token as *mother* than *child*. Finally, the tokens classified as *father* by the ASP system were approximately twice as likely to be classified as *other* by human coders than were either *child* or *mother* ASP-system-classifications.

**Fig 2:**
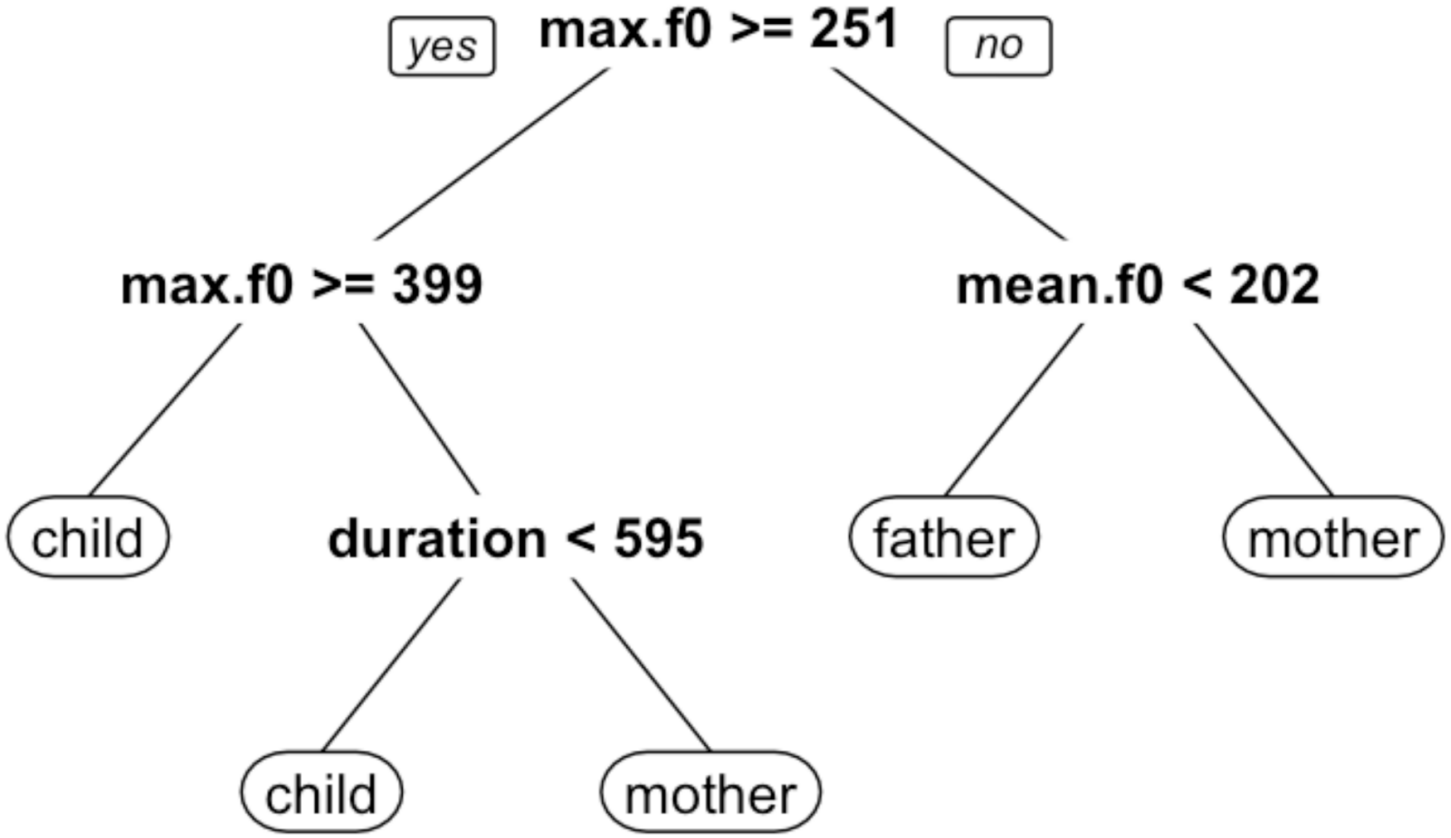
Classification tree fit to ASP system judgments. Nodes indicate best fitting partitions for acoustic variables. The model implements a decision procedure starting from the top of the figure and proceeding downwards.

The classification tree fit to the ASP system’s judgments is illustrated in Fig 2. The illustrated trees depict a decision procedure wherein a stimulus is evaluated according to the stated inequalities in each node, beginning at the top and descending until a terminal node is reached. So, for example, the classification tree fit to the ASP system judgments begins by evaluating the maximum *f*_0_ of a given stimulus. If the maximum *f*_0_ is greater than or equal to 251 Hz, the left branch is followed. If the maximum *f*_0_ is greater than or equal to 399 Hz, the stimulus is classified as *child*, whereas if it is less than 399 Hz, the duration of the stimulus is evaluated. If the duration is less than 595 ms, the stimulus is classified at *child*, whereas if it is greater than or equal to 595 ms, it is classified as *mother*. If the maximum *f*_0_ is less than 251 at the first node, the mean *f*_0_ is evaluated. If the mean *f*_0_ is less than 202 Hz, the stimulus is classified as *father*, otherwise it is classified at *mother*. A ten-fold cross-validation was performed on the classification tree fit of the machine decisions. In this process, 90% of the data was used to train the classification tree model, with an error term computed on the held-out 10%. This process was repeated with ten arbitrary, unique training-error sets. The overall cross-validation error rate for this data set is 0.124.

**Fig 3:**
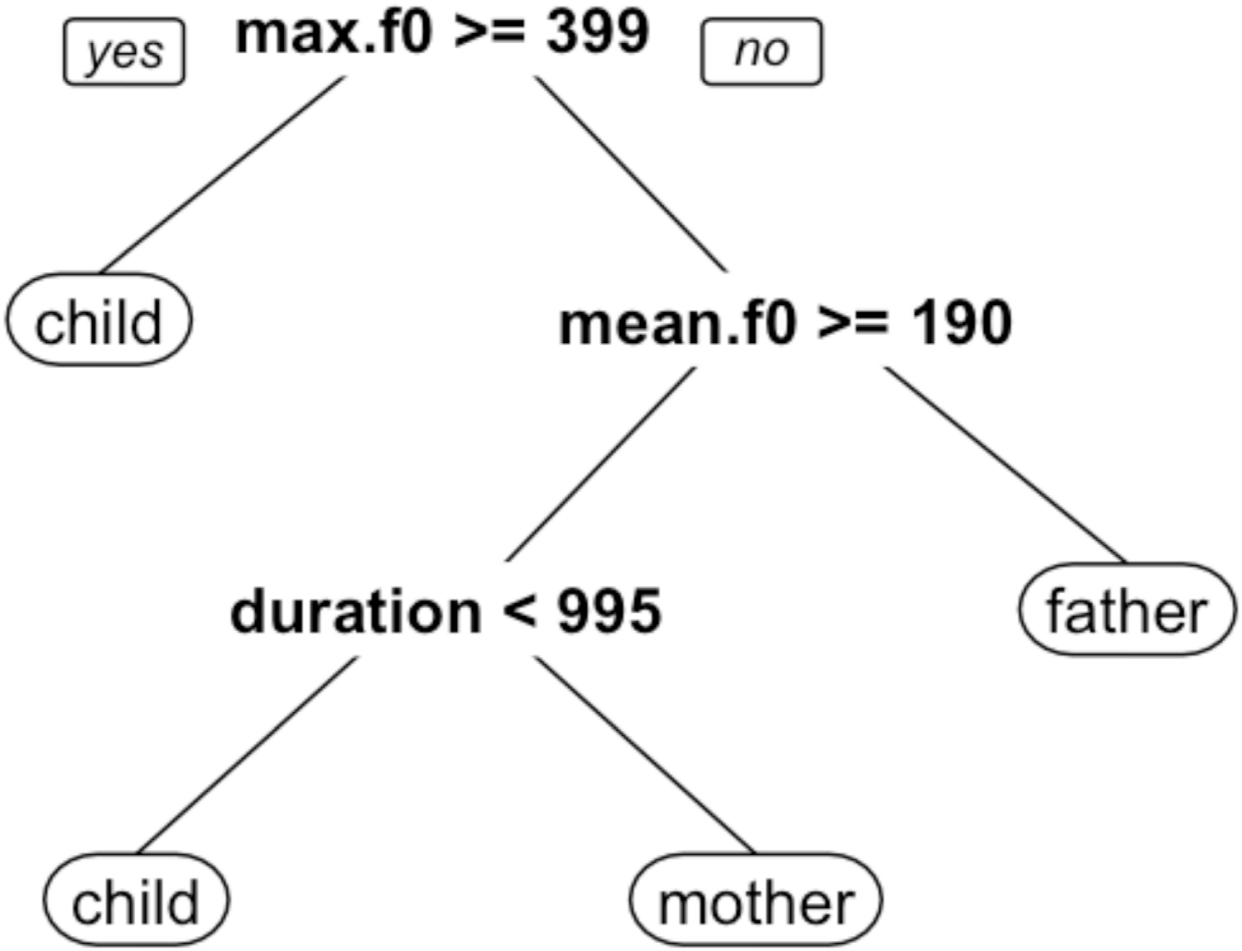
Classification tree fit to human coder judgments. Nodes indicate best fitting partitions for acoustic variables. The model implements a decision procedure starting from the top of the figure and proceeding downwards.

The classification tree fit to the human coder’s judgments is illustrated in Fig 3. In this tree, the decision procedure also begins by evaluating the maximum *f*_0_ of a given stimulus. If the maximum *f*_0_ is greater than or equal to 399 Hz, the stimulus is classified as *child*. If the maximum f0 is less than 399 Hz, the mean *f*_0_ is evaluated. If the mean *f*_0_ is less than 190 Hz, the stimulus is classified as *father*, whereas if the mean *f*_0_ is greater than or equal to 190 Hz, the duration of the stimulus is evaluated. If the duration is less than 995 ms, the stimulus is classified as *child*, otherwise it is classified as *mother*. The same ten-fold cross-validation procedure described above was performed on the classification tree fit of the human decisions, except decisions from all 23 judges were entered into the model (thus, the data was about 23-times larger for this dataset). The overall cross-validation error rate for this data set is 0.353.

## Discussion

This work examined the accuracy of ASP labels in a large sample of speech collected from natural family settings using LENA technology. This work is motivated by a need to establish the accuracy of ASP technology in common use in the current research literature and clinical practice. We found a high degree of agreement between the ASP system and human coders, as indicated by high unweighted Fleiss’ and Cohen’s kappa coefficients and moderate-to-high weighted kappa coefficients [42].

Consideration of the pattern of disagreements between the ASP system and human coders indicates an asymmetry on the part of the ASP system relative to human coders. When the ASP system and human coders disagreed, human *child* classifications were mostly classified as *mother* by the ASP system, whereas human *mother* classifications were more often classified as *father* by the ASP system, as were human *father* classifications.

Analysis of the relationships between ten acoustic measures and the observed classifications indicate that both the ASP system’s and the human coders’ judgments correspond most closely to differences in maximum *f*_0_, mean *f*_0_, and duration. The classification tree models fit to the ASP and human coder judgments are very similar, both indicating that tokens classified as *child* exhibited high (maximum and/or mean) *f*_0_ values and shorter durations, while tokens classified as *father* exhibited low (maximum and/or mean) *f*_0_ values, and tokens classified as *mother* exhibited moderate to high *f*_0_ values and longer durations. Taken as a whole, these findings show certain structural similarities between the human decision processes known to be important for speech perception (namely, *f*_0_, amplitude, and temporal characteristics) and the results of the machine algorithms.

Overall, the machine performance found here is consistent with ASP performance [43,44]given the naturalistic, open-set acoustic data the system takes as input.

### Practical application

Results from this work could be used to improve the algorithms and ASP procedures generating output. This is certainly not a straightforward task and the current results give rather abstract areas for improvement. Future work might explore concrete methods for improving the technology. For example, human classification decisions are demonstrated here to be influenced by spectral and temporal aspects of the acoustic signal with little influence from amplitude characteristics. This finding might guide researchers to focus on parameters likely to be useful for human applications such as speech. Another application might use the results of the present work directly to interpret future application of the ASP output. In particular, this work provides a fairly detailed estimate of the error (broadly defined) of the system output. This error, detailed by the label types examined here, could be straightforwardly interpreted alongside the input to better understand the results. For example, error estimates of the label outputs could be input into a model as the likelihood that a given label is correctly assigned, a sort of confidence coefficient for the classification results. This work is a first step in that direction, giving likelihood estimates for labels most likely to be useful for speech research, namely the target child, adult female, and adult male vocalizations.

### Limitations

We do not account for segmentation in this work, but instead simply assume that segmentation is meaningful. It is unknown if changes or improvements in segmentation would alter the performance of the ASP or the judges.

Differences between the ASP labels (*FAN*, *MAN*, *CHN*) and the choices presented to judges (*mother*, *father*, *child*) are not necessarily commensurate. The LENA labels are intended to indicate a relatively high similarity between the model for adult female, adult male, or the key child wearing the device, respectively, and the sampled audio segment. The LENA model does not assume the segment bears a specific relationship such as father or mother.

The specific acoustic features used in this study could be profitably expanded. Although this study was designed to examine relatively coarse features due to the preliminary nature of this investigation and the broad variability in labels ostensibly identifying 78 individuals (mothers, fathers, and children in 26 families). Other work, notably Oller and colleagues [14], examined the role of 12 acoustic features from four general speech categories (the rhythmic/syllabification group, the low spectral tilt and high pitch control group, the wide formant bandwidth and low pitch control group, and the duration group) known to be relevant for child vocalizations. Examining the automatically coded vocalizations of children, they showed associations between the apriori acoustic features/feature classes and group classifications into typical and disordered classes. Future work could benefit from a wider application of acoustic features to better understand the underlying mechanism of classification for talker or group classification.

The ASP and human judges make decisions based on different factors. Despite conclusions that may be equitable or interpretable in terms of the other, it is not clear how much insight the ASP offers into the human decision process. For example, human judges certainly used semantic content in the decision process, a detail inaccessible to the ASP. The ASP may have also make use of details such as detailed representations of energy in the signal that may not be used in the same way for human listeners. Similarly, there is no guarantee that the acoustic factors consider here or in other post-hoc analyses such as Oller and colleagues [14] have analogs to those used by the inaccessible processing algorithms of the LENA system. Unless or until those processing techniques are made transparent, the acoustic correlates described here are, at best, secondary to the actual system performance.

## References

[1] Eskanazi M. An overview of spoken language technology for education. Speech Communication. 2009;51:832–844. 10.1016/j.specom.2009.04.005

[2] Potamianos A, Narayanan S, Lee S. Automatic speech recognition for children. 1997. Paper presented at the Fifth European Conference on Speech Communication and Technology, EUROSPEECH. Rhodes, Greece.

[3] Das S, Nix D, Picheny M. Improvements in Children's Speech Recognition Performance. Proceedings of the 1998 IEEE International Conference on Acoustics, Speech, and Signal Processing. 1998;1:433–436.

[4] Gerosa M, Giuliani D, Narayanan S, Potamianos A. A review of ASR technologies for children's speech. Proc 2nd Workshop Child, Computer and Interaction, ACM 2009 Nov 05: 1–7.

[5] Giuliani D, Gerosa, M. Investigating recognition of children's speech. Proc IEEE International Conference on Acoustics, Speech, and Signal Processing. 2003;2:II-137.

[6] Gerosa M, Giuliani D, Brugnara F. Acoustic Variability and Automatic Recognition of Children’s Speech. Speech Communication, 2007;49(10): 847–860. pii:S0167-6393(07)00005-2 10.1016/j.specom.2007.01.002

[7] Russell M, Brown C, Skilling A, Series R, Wallace J, Bonham B, et al. Applications of automatic speech recognition to speech and language development in young children. Proc 4th IEEE International Conference on Spoken Language Processing. 1996 Oct: 176–179.

[8] Meilleur AA, and Fombonne E. Regression of language and non‐language skills in pervasive developmental disorders. J Intellect Disabil Res. 2009;53(2): 115–124. 10.1111/j.1365-2788.2008.01134.x 19054269

[9] Levitt H. Processing of speech signals for physical and sensory disabilities. Proc Natl Adac Sci U S A. 1995;92: 9999–100006. 10.1073/pnas.92.22.9999 PMC40725 7479816

[10] VanDam M. Acoustic characteristics of the clothes used for a wearable recording device. J Acoust Soc Am. 2014; 136(4): EL263–EL267. 10.1121/1.4895015 25324108

[11] VanDam M, Warlaumont AS, Bergleson E, Cristia A, Soderstrom M, De Palma P, et al HomeBank, an online repository of daylong child-centered audio recordings. Sem Speech Lang. 2016;37:128–142. 10.1055/s00036-1580745 PMC5570530 27111272

[12] Zimmerman FJ, Gilkerson J, Richards JA, Christakis DA, Xu D, Gray S, et al Teaching by listening: The importance of adult-child conversations to language development. Pediatrics. 2009;124:342–349. 10.1542/peds.2008-2267 19564318

[13] Gilkerson J, Richards JA. The power of talk: Impact of adult talk, conversational turns, and TV during the critical 0–4 years of child development (Technical Report LTR-01-2, 2nd ed.). 2009. Boulder, CO: LENA Foundation. Available: www.lenafoundation.org/wp-content/uploads/2014/10/LTR-01-2_PowerOfTalk.pdf. Accessed 01 May 2015.

[14] Oller DK, Niyogi P, Gray S, Richards JA, Gilkerson J, Xu D, et al Automated vocal analysis of naturalistic recordings from children with autism, language delay, and typical development. Proc Natl Adac Sci U S A. 2010;107(30): 13354–13359. 10.1073/pnas.1003882107 PMC2922144 20643944

[15] Warren SF, Gilkerson J, Richards JA, Oller DK, Xu D, Yapanel U, et al What automated vocal analysis reveals about the vocal production and language learning environment of young children with Autism. J Autism Dev Disord. 2010;40(5): 555–569. 10.1007/s10803-009-0902-5 19936907

[16] Dykstra JR, Sabatos-DeVito MG, Irvin DW, Boyd BA, Hume KA, Odom SL. Using Language Environment Analysis (LENA) system in preschool classrooms with children with autism spectrum disorder. Autism. 2013;17(5):582–594. 10.1177/1362361312446206 22751753

[17] Warlaumont AS, Richards JA, Gilkerson J, Oller DK. A social feedback loop for speech development and its reduction in autism. Psychological Science. 2014;25: 1314–1324. 10.1177/0956797614531023 24840717 PMC4237681

[18] VanDam M, Oller DK, Ambrose SE, Gray S, Richards JA, Xu D, et al Automated vocal analysis of children with hearing loss and their typical and atypical peers. Ear Hear. 2015;36(4): e146–e152. 25587667 10.1097/AUD.0000000000000138 PMC4478108

[19] VanDam M, Ambrose SE, Moeller MP. Quantity of parental language in the home environments of hard-of-hearing 2-year-olds. J Deaf Stud Deaf Educ. 2012;17:402–420. 10.1093/deafed/ens025 22942314 PMC3529623

[20] VanDam M, Moeller MP, Tomblin B. Analyses of fundamental frequency in infants and preschoolers with hearing loss. J Acoust Soc Am. 2010;128(4:2): 2459.

[21] Theimann-Bourque KS, Warren SF, Brady N, Gilkerson J, Richards JA. Vocal interaction between children with Down Syndrome and their parents. Am J Speech Lang Pathol. 2014;23: 474–485. 10.1044/2014_AJSLP-12-0010 24686777 PMC4257479

[22] Caskey M, Stephens B, Tucker R, Vohr B. Importance of parent talk on the development of preterm infant vocalizations. Pediatrics. 2011;218(5): 910–916. 10.1542/peds.2011-0609 22007020

[23] Johnson K, Caskey M, Rand K, Tucker R, Vohr B. Gender differences in adult-infant communication in the first months of life. Pediatrics. 2014;134(6): e1603–e1610. 10.1542/peds.2013-4289 25367542

[24] Christakis DA, Gilkerson J, Richards JA, Zimmerman FJ, Garrison MM, Xu D, et al Audible television and decreased adult words, infant vocalizations, and conversational turns. Arch Pediatr Adolesc Med. 2009;163(6): 554–558. 10.1001/archpediatrics.2009.61 19487612

[25] Ambrose SE, VanDam M, Moeller MP. Linguistic input, electronic media, and communication outcomes in toddlers with hearing loss. Ear Hear. 2014;35(2): 139–147. 24441740 10.1097/AUD.0b013e3182a76768 PMC3944057

[26] Aragon M, Yoshinaga-Itano C. Using Language Environment Analysis to improve outcomes for children who are deaf or hard of hearing. Sem Speech Lang. 2012;33(4): 340–353. 10.1055/s-0032-1326918 23081793

[27] Wang Z, Pan X, Miller KF, Cortina KS. Automatic classification of activities in classroom discourse. Comput Educ. 2014;78: 115–123. 10.1016/j.compedu.2014.05.010

[28] Xu D, Yapanel U, Gray S. Reliability of the LENA Language Environment Analysis System in young children’s natural home environment (Technical Report LTR-05-2). Boulder, CO: LENA Foundation. 2009. Available: www.lenafoundation.org/TechReport.aspx/Reliability/LTR-05-2. Accessed 20 November, 2014.

[29] Paul T, Xu D, Richards JA. System and method for expressive language assessment. 2014. Patent Number US 8844847 B2. Available: http://www.google.com.ar/patents/US874484. Accessed 21 July 2014.

[30] Xu D, Yapanel U, Gray S, Gilkerson J, Richards JA, Hansen J. Signal processing for young child speech language development. 2008. Paper presented at The 1st workshop on child, computer and interaction, Chania, Crete, Greece.

[31] Xu D, Yapanel U, Gray S, Baer CT. The LENATM language environment analysis system: The interpretive time segments (ITS) file (LENA Foundation Technical Report LTR-04-2). 2008. Available: http://www.lenafoundation.org/TechReport.aspx/ITS_File/LTR-04-2. Accessed 14 January 2014.

[32] Bořil H, Zhang Q, Ziaei A, Hansen JHL, Xu D, Gilkerson J, et al. Automatic assessment of language background in toddlers through phonotactic and pitch pattern modeling of short vocalizations. Fourth Workshop on Child, Computer and Interaction. 2014. Available: http://www.utd.edu/~hynek/pdfs/WOCCI14.pdf. Accessed 20 July 2015.

[33] Xu D, Richards JA, Gilkerson J. Automated analysis of child phonetic production using naturalistic recordings. J Speech Lang Hear Res. 2014;57: 1638–1650. 10.1044/2014_JSLHR-S-13-0037 24824489

[34] Soderstrom M, Wittebolle K. When do caregivers talk? The influences of activity and time of day on caregiver speech and child vocalizations in two childcare environments. PLoS ONE. 2013;8: e80646 10.1371/journal.pone.0080646 24260443 PMC3832484

[35] Weisleder A, Fernald A. Talking to children matters: Early language experience strengthens processing and builds vocabulary. Psychological Science. 2013;24: 2143–2152. 24022649 10.1177/0956797613488145 PMC5510534

[36] Canault M, LeNormand M-T, Foudil S, Loundon N, Thai-Van H. Reliability of the Language ENvironment Analysis system (LENA TM) in European French. Behav Res. 2015; Available: http://link.springer.com/article/10.3758/s13428-015-0634-8. Accessed 24 July 2015. 10.3758/s13428-015-0634-8

[37] Oller DK. The Emergence of the Speech Capacity. Mahway, NJ: Erlbaum 2000.

[38] Stevens KN. Toward a model for lexical access based on acoustic landmarks and distinctive features. J Acoust Soc Am. 2002;111(4): 1872–1891. 12002871 10.1121/1.1458026

[39] Milborrow S. rpart.plot: Plot rpart Models. An Enhanced Version of plot.rpart. R package version 1.5.2. 2015. Available: http://CRAN.R-project.org/package=rpart.plot. Accessed 01 May 2015.

[40] R Core Team. R: A language and environment for statistical computing. R Foundation for Statistical Computing, Vienna, Austria 2014 Available: http://www.R-project.org/. Accessed 01 May 2015.

[41] Therneau T, Atkinson B, Ripley B. rpart: Recursive Partitioning and Regression Trees. R package version 4.1–8. 2014. Available: http://CRAN.R-project.org/package=rpart. Accessed 01 Feb 2015.

[42] Fleiss JL. Statistical methods for rates and proportions. 1981 New York: John Wiley.

[43] Gray SS, Willett D, Lu J, Pinto J, Maergner P, Bodenstab N. Child automatic speech recognition for US English: Child interaction with living-room-electronic-devices. Paper presented at the Fifth Workshop on Child Computer Interaction WOCCI. 2014. San Francisco, CA.

[44] Cheng J, Chen X, Metallinou A. Deep neural network acoustic models for spoken assessment applications. Speech Communication. 2015;73:14–27.

